# *tert*-Butyl *N*-benzyl-*N*-(4-methyl-2-pyrid­yl)carbamate

**DOI:** 10.1107/S1600536808034491

**Published:** 2008-10-31

**Authors:** Pierre Koch, Dieter Schollmeyer, Stefan Laufer

**Affiliations:** a Institute of Pharmacy, Department of Pharmaceutical and Medicinal Chemistry, Eberhard-Karls-University Tübingen, Auf der Morgenstelle 8, 72076 Tübingen, Germany; b Department of Organic Chemistry, Johannes Gutenberg-University Mainz, Duesbergweg 10-14, D-55099 Mainz, Germany

## Abstract

In the crystal structure of the title compound, C_18_H_22_N_2_O_2_, the pyridine ring makes dihedral angles of 83.71 (6) and 9.2 (1)° with the phenyl ring and the carbamate plane, respectively. The phenyl ring and the carbamate plane are nearly perpendicular to one another, with a dihedral angle of 87.17 (7)°.

## Related literature

For the preparation of the title compound, see: Koch *et al.* (2008 ▶). For applications of *N*-benzyl-2-amino­pyridines, see, for example: Laufer & Koch (2008 ▶); Koch *et al.* (2008 ▶); Lipinski *et al.* (1985 ▶); Miwatashi *et al.* (2005 ▶); Stevens *et al.* (2005 ▶).
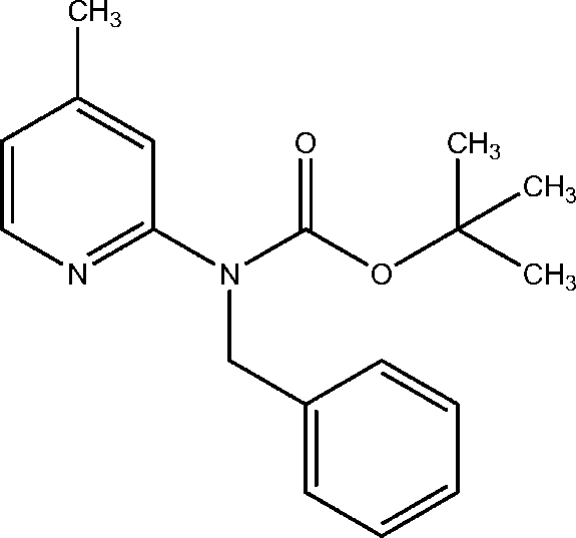


## Experimental

### 

#### Crystal data

C_18_H_22_N_2_O_2_
*M* *_r_* = 298.38
Triclinic, 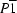

*a* = 5.9090 (10) Å
*b* = 9.7779 (18) Å
*c* = 14.199 (7) Å
α = 89.683 (13)°
β = 87.968 (14)°
γ = 83.963 (15)°
*V* = 815.3 (5) Å^3^
*Z* = 2
Cu *K*α radiation
μ = 0.63 mm^−1^
*T* = 193 (2) K
0.45 × 0.45 × 0.33 mm

#### Data collection

Enraf–Nonius CAD-4 diffractometer
Absorption correction: none
5914 measured reflections
3074 independent reflections
2747 reflections with *I* > 2σ(*I*)
*R*_int_ = 0.090
3 standard reflections frequency: 60 min intensity decay: 3%

#### Refinement

*R*[*F* ^2^ > 2σ(*F* ^2^)] = 0.055
*wR*(*F* ^2^) = 0.209
*S* = 1.12
3074 reflections
204 parameters
H-atom parameters constrained
Δρ_max_ = 0.31 e Å^−3^
Δρ_min_ = −0.37 e Å^−3^

### 

Data collection: *CAD-4 Software* (Enraf–Nonius, 1989 ▶); cell refinement: *CAD-4 Software*; data reduction: *CORINC* (Dräger & Gattow, 1971 ▶); program(s) used to solve structure: *SIR97* (Altomare *et al.*, 1999 ▶); program(s) used to refine structure: *SHELXL97* (Sheldrick, 2008 ▶); molecular graphics: *PLATON* (Spek, 2003 ▶); software used to prepare material for publication: *PLATON*.

## Supplementary Material

Crystal structure: contains datablocks I, global. DOI: 10.1107/S1600536808034491/zl2149sup1.cif

Structure factors: contains datablocks I. DOI: 10.1107/S1600536808034491/zl2149Isup2.hkl

Additional supplementary materials:  crystallographic information; 3D view; checkCIF report

## Figures and Tables

**Fig. 1.**
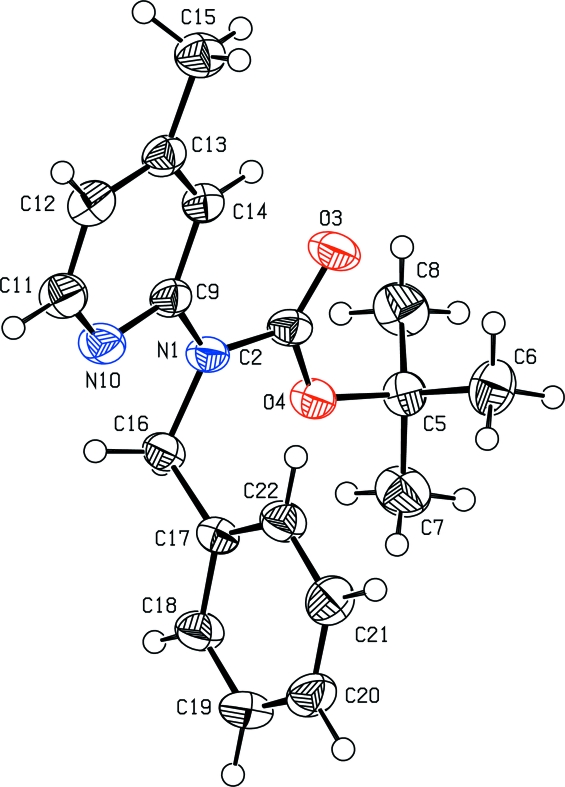
View of compound I. Displacement ellipsoids are drawn at the 50% probability level. H atoms are depicted as circles of arbitrary size.

## supplementary crystallographic information

### Comment

*N*-Benzyl-2-aminopyridin-4-yl derivatives can be found in different p38 MAP kinase inhibitors, like the imidazolopyridines (Laufer & Koch 2008; Koch *et al.* 2008), thiazolopyridines (Miwatashi *et al.* 2005) or pyrazolopyridines (Stevens *et al.* 2005) and in histamine H_2_-receptor antagonists (Lipinski *et al.* 1985).

The title compound, *tert*-butyl *N*-benzyl-*N*-(4-methylpyridin-2-yl)carbamate (I), was obtaineded as an intermediate in the synthesis of 2-alkylsulfanyl-5-(2-aminopyridin-4-yl)-4-(4-fluorophenyl)imidazoles as potent p38 MAP kinase inhibitors (Laufer & Koch 2008; Koch *et al.* 2008).

In the crystal structure of the title compound I the pyridine ring makes dihedral angles of 83.71 (6)° and 9.2 (1)° to the phenyl ring and the carbamate plane, respectively. The phenyl ring and the carbamate plane are nearly perpendicular to one another with a dihedral angle of 87.17 (7)°. The N1—C2 bond [1.383 (2) Å] of the carbamte function is shorter than the normal N1—C16-bond [1.475 (2) Å] to the benzyl moiety, indicating the partial double bond character of the amide bond of the carbamate.

### Experimental

To a solution of *tert*-butyl 4-methylpyridin-2-ylcarbamate (0.75 g, 3.6 mmol) in dry DMF (11 ml) was added under an argon-atmosphere sodium hydride (0.18 g, 4.5 mmol, 60% oil dispersion) at 273 K in such a manner that the temperature was kept below 278 K. The reaction mixture was kept at 273 K for 20 min followed by the addition of benzyl bromide (0.71 g, 4.1 mmol) at the same temperature. After additional stirring at 273 K for 30 min the mixture was allowed to warm to room temperature within 1 h, after which water and ethyl acetate were added. The organic layer was washed subsequently with HCl (0.1 *M*), sodium bicarbonate and brine, dried (sodium sulfate) and concentrated *in vacuo*. The residue was purified by flash-chromatography (silica gel, *n*-hexane/ethyl acetate 3:1) to yield 0.60 g (56%) of I as a colourless solid (Koch *et al.* 2008). Recrystallization from hot *n*-hexane/ethyl acetate afforded colourless crystals.

### Refinement

Hydrogen atoms attached to carbons were placed at calculated positions with C—H = 0.95 Å (aromatic) or 0.98–0.99 Å (*sp*^3^ C-atom). All H atoms were refined in the riding-model approximation with isotropic displacement parameters (set at 1.2–1.5 times of the *U*_eq_ of the parent atom).

### Crystal data

**Table:**

C_18_H_22_N_2_O_2_	*Z* = 2
*M**_r_* = 298.38	*F*(000) = 320
Triclinic, *P*1	*D*_x_ = 1.215 Mg m^−^^3^
Hall symbol: -P 1	Cu *K*α radiation, λ = 1.54178 Å
*a* = 5.909 (1) Å	Cell parameters from 25 reflections
*b* = 9.7779 (18) Å	θ = 65–70°
*c* = 14.199 (7) Å	µ = 0.63 mm^−^^1^
α = 89.683 (13)°	*T* = 193 K
β = 87.968 (14)°	Block, yellow
γ = 83.963 (15)°	0.45 × 0.45 × 0.33 mm
*V* = 815.3 (5) Å^3^	

### Data collection

**Table:**

Enraf–Nonius CAD-4 diffractometer	*R*_int_ = 0.090
Radiation source: rotating anode	θ_max_ = 69.9°, θ_min_ = 3.1°
graphite	*h* = −7→7
ω/2θ scans	*k* = −11→11
5914 measured reflections	*l* = −17→17
3074 independent reflections	3 standard reflections every 60 min
2747 reflections with *I* > 2σ(*I*)	intensity decay: 3%

### Refinement

**Table:**

Refinement on *F*^2^	Secondary atom site location: difference Fourier map
Least-squares matrix: full	Hydrogen site location: inferred from neighbouring sites
*R*[*F*^2^ > 2σ(*F*^2^)] = 0.055	H-atom parameters constrained
*wR*(*F*^2^) = 0.209	*w* = 1/[σ^2^(*F*_o_^2^) + (0.1082*P*)^2^ + 0.2701*P*] where *P* = (*F*_o_^2^ + 2*F*_c_^2^)/3
*S* = 1.12	(Δ/σ)_max_ < 0.001
3074 reflections	Δρ_max_ = 0.31 e Å^−^^3^
204 parameters	Δρ_min_ = −0.37 e Å^−^^3^
0 restraints	Extinction correction: SHELXL97 (Sheldrick, 2008), Fc^*^=kFc[1+0.001xFc^2^λ^3^/sin(2θ)]^-1/4^
Primary atom site location: structure-invariant direct methods	Extinction coefficient: 0.034 (4)

### Special details

**Table:**

Geometry. All e.s.d.'s (except the e.s.d. in the dihedral angle between two l.s. planes) are estimated using the full covariance matrix. The cell e.s.d.'s are taken into account individually in the estimation of e.s.d.'s in distances, angles and torsion angles; correlations between e.s.d.'s in cell parameters are only used when they are defined by crystal symmetry. An approximate (isotropic) treatment of cell e.s.d.'s is used for estimating e.s.d.'s involving l.s. planes.
Refinement. Refinement of *F*^2^ against ALL reflections. The weighted *R*-factor *wR* and goodness of fit *S* are based on *F*^2^, conventional *R*-factors *R* are based on *F*, with *F* set to zero for negative *F*^2^. The threshold expression of *F*^2^ > σ(*F*^2^) is used only for calculating *R*-factors(gt) *etc*. and is not relevant to the choice of reflections for refinement. *R*-factors based on *F*^2^ are statistically about twice as large as those based on *F*, and *R*-factors based on ALL data will be even larger.

### Fractional atomic coordinates and isotropic or equivalent isotropic displacement parameters (Å^2^)

**Table:**

	*x*	*y*	*z*	*U*_iso_*/*U*_eq_	
N1	0.6712 (3)	0.71560 (15)	0.31250 (11)	0.0319 (4)	
C2	0.6184 (3)	0.78127 (18)	0.22834 (14)	0.0333 (5)	
O3	0.7015 (3)	0.88142 (15)	0.19760 (11)	0.0464 (4)	
O4	0.4607 (2)	0.71849 (14)	0.18563 (10)	0.0359 (4)	
C5	0.3936 (3)	0.7575 (2)	0.08933 (14)	0.0347 (5)	
C6	0.6005 (4)	0.7399 (3)	0.02209 (16)	0.0487 (6)	
H6A	0.7010	0.8107	0.0348	0.073*	
H6B	0.5510	0.7488	−0.0430	0.073*	
H6C	0.6829	0.6487	0.0311	0.073*	
C7	0.2306 (4)	0.6529 (3)	0.06899 (18)	0.0539 (6)	
H7A	0.1826	0.6636	0.0038	0.081*	
H7B	0.0970	0.6673	0.1120	0.081*	
H7C	0.3068	0.5600	0.0780	0.081*	
C8	0.2739 (4)	0.9025 (2)	0.08803 (19)	0.0518 (6)	
H8A	0.3828	0.9681	0.1016	0.078*	
H8B	0.1497	0.9108	0.1359	0.078*	
H8C	0.2119	0.9222	0.0257	0.078*	
C9	0.8500 (3)	0.75070 (19)	0.36948 (13)	0.0321 (5)	
N10	0.8999 (3)	0.66158 (17)	0.43875 (12)	0.0392 (5)	
C11	1.0670 (4)	0.6864 (2)	0.49585 (15)	0.0422 (5)	
H11	1.1012	0.6238	0.5460	0.051*	
C12	1.1917 (3)	0.7972 (2)	0.48605 (15)	0.0405 (5)	
H12	1.3094	0.8102	0.5280	0.049*	
C13	1.1410 (3)	0.8897 (2)	0.41319 (14)	0.0352 (5)	
C14	0.9660 (3)	0.86750 (19)	0.35502 (13)	0.0337 (5)	
H14	0.9247	0.9305	0.3059	0.040*	
C15	1.2734 (3)	1.0119 (2)	0.39721 (16)	0.0434 (5)	
H15A	1.2841	1.0599	0.4570	0.065*	
H15B	1.1955	1.0747	0.3519	0.065*	
H15C	1.4268	0.9805	0.3723	0.065*	
C16	0.5592 (3)	0.59215 (19)	0.33849 (14)	0.0331 (5)	
H16A	0.3957	0.6093	0.3245	0.040*	
H16B	0.5700	0.5772	0.4073	0.040*	
C17	0.6591 (3)	0.46264 (18)	0.28813 (13)	0.0306 (4)	
C18	0.5344 (3)	0.3499 (2)	0.28782 (15)	0.0404 (5)	
H18	0.3877	0.3563	0.3181	0.048*	
C19	0.6209 (4)	0.2281 (2)	0.24390 (18)	0.0492 (6)	
H19	0.5331	0.1521	0.2441	0.059*	
C20	0.8339 (4)	0.2171 (2)	0.19997 (16)	0.0463 (6)	
H20	0.8937	0.1338	0.1700	0.056*	
C21	0.9597 (4)	0.3287 (2)	0.20003 (16)	0.0449 (5)	
H21	1.1061	0.3220	0.1695	0.054*	
C22	0.8739 (3)	0.4502 (2)	0.24424 (15)	0.0382 (5)	
H22	0.9629	0.5256	0.2445	0.046*	

### Atomic displacement parameters (Å^2^)

**Table:**

	*U*^11^	*U*^22^	*U*^33^	*U*^12^	*U*^13^	*U*^23^
N1	0.0366 (8)	0.0237 (8)	0.0358 (9)	−0.0040 (6)	−0.0036 (6)	0.0012 (6)
C2	0.0361 (9)	0.0232 (9)	0.0401 (10)	−0.0003 (7)	−0.0008 (7)	−0.0005 (7)
O3	0.0612 (9)	0.0325 (8)	0.0488 (9)	−0.0166 (6)	−0.0133 (7)	0.0106 (7)
O4	0.0377 (7)	0.0326 (7)	0.0384 (8)	−0.0070 (5)	−0.0082 (5)	0.0039 (6)
C5	0.0322 (9)	0.0366 (11)	0.0349 (10)	−0.0008 (7)	−0.0054 (7)	0.0024 (8)
C6	0.0409 (11)	0.0610 (14)	0.0431 (12)	−0.0011 (9)	0.0011 (9)	−0.0060 (10)
C7	0.0515 (12)	0.0581 (15)	0.0557 (14)	−0.0194 (11)	−0.0143 (10)	0.0072 (11)
C8	0.0484 (11)	0.0431 (13)	0.0608 (14)	0.0107 (9)	−0.0046 (10)	0.0099 (11)
C9	0.0365 (9)	0.0251 (9)	0.0336 (10)	0.0014 (7)	−0.0004 (7)	−0.0021 (7)
N10	0.0486 (9)	0.0289 (9)	0.0402 (9)	−0.0023 (7)	−0.0088 (7)	0.0047 (7)
C11	0.0506 (11)	0.0353 (11)	0.0405 (11)	−0.0003 (8)	−0.0120 (9)	0.0034 (9)
C12	0.0399 (10)	0.0392 (11)	0.0419 (11)	−0.0002 (8)	−0.0060 (8)	−0.0039 (9)
C13	0.0336 (9)	0.0324 (10)	0.0387 (10)	−0.0003 (7)	0.0023 (7)	−0.0060 (8)
C14	0.0379 (9)	0.0290 (9)	0.0340 (10)	−0.0026 (7)	−0.0004 (7)	−0.0013 (8)
C15	0.0376 (10)	0.0429 (12)	0.0509 (12)	−0.0095 (8)	−0.0014 (8)	−0.0022 (10)
C16	0.0338 (9)	0.0273 (9)	0.0380 (10)	−0.0042 (7)	0.0030 (7)	0.0027 (8)
C17	0.0316 (8)	0.0256 (9)	0.0351 (9)	−0.0043 (7)	−0.0032 (7)	0.0043 (7)
C18	0.0398 (10)	0.0342 (10)	0.0486 (12)	−0.0117 (8)	0.0006 (8)	0.0005 (9)
C19	0.0628 (13)	0.0295 (11)	0.0572 (14)	−0.0143 (9)	−0.0015 (10)	0.0012 (10)
C20	0.0620 (13)	0.0291 (10)	0.0458 (12)	0.0055 (9)	−0.0047 (10)	−0.0037 (9)
C21	0.0407 (10)	0.0443 (12)	0.0478 (12)	0.0025 (8)	0.0027 (9)	−0.0048 (10)
C22	0.0359 (9)	0.0337 (10)	0.0454 (12)	−0.0068 (7)	0.0041 (8)	0.0002 (9)

### Geometric parameters (Å, °)

**Table:**

N1—C2	1.383 (3)	C12—C13	1.390 (3)
N1—C9	1.424 (3)	C12—H12	0.9500
N1—C16	1.475 (2)	C13—C14	1.381 (3)
C2—O3	1.213 (2)	C13—C15	1.507 (3)
C2—O4	1.333 (2)	C14—H14	0.9500
O4—C5	1.474 (2)	C15—H15A	0.9800
C5—C7	1.512 (3)	C15—H15B	0.9800
C5—C8	1.516 (3)	C15—H15C	0.9800
C5—C6	1.520 (3)	C16—C17	1.512 (3)
C6—H6A	0.9800	C16—H16A	0.9900
C6—H6B	0.9800	C16—H16B	0.9900
C6—H6C	0.9800	C17—C22	1.388 (3)
C7—H7A	0.9800	C17—C18	1.389 (3)
C7—H7B	0.9800	C18—C19	1.388 (3)
C7—H7C	0.9800	C18—H18	0.9500
C8—H8A	0.9800	C19—C20	1.379 (3)
C8—H8B	0.9800	C19—H19	0.9500
C8—H8C	0.9800	C20—C21	1.383 (3)
C9—N10	1.331 (3)	C20—H20	0.9500
C9—C14	1.403 (3)	C21—C22	1.386 (3)
N10—C11	1.342 (3)	C21—H21	0.9500
C11—C12	1.377 (3)	C22—H22	0.9500
C11—H11	0.9500		
			
C2—N1—C9	122.86 (15)	C11—C12—H12	120.9
C2—N1—C16	118.75 (16)	C13—C12—H12	120.9
C9—N1—C16	117.88 (15)	C14—C13—C12	118.64 (18)
O3—C2—O4	124.64 (19)	C14—C13—C15	120.26 (18)
O3—C2—N1	125.50 (18)	C12—C13—C15	121.11 (19)
O4—C2—N1	109.86 (15)	C13—C14—C9	119.18 (18)
C2—O4—C5	120.92 (14)	C13—C14—H14	120.4
O4—C5—C7	101.60 (15)	C9—C14—H14	120.4
O4—C5—C8	110.50 (17)	C13—C15—H15A	109.5
C7—C5—C8	111.09 (18)	C13—C15—H15B	109.5
O4—C5—C6	109.95 (15)	H15A—C15—H15B	109.5
C7—C5—C6	110.71 (19)	C13—C15—H15C	109.5
C8—C5—C6	112.47 (17)	H15A—C15—H15C	109.5
C5—C6—H6A	109.5	H15B—C15—H15C	109.5
C5—C6—H6B	109.5	N1—C16—C17	114.19 (14)
H6A—C6—H6B	109.5	N1—C16—H16A	108.7
C5—C6—H6C	109.5	C17—C16—H16A	108.7
H6A—C6—H6C	109.5	N1—C16—H16B	108.7
H6B—C6—H6C	109.5	C17—C16—H16B	108.7
C5—C7—H7A	109.5	H16A—C16—H16B	107.6
C5—C7—H7B	109.5	C22—C17—C18	118.39 (18)
H7A—C7—H7B	109.5	C22—C17—C16	122.65 (16)
C5—C7—H7C	109.5	C18—C17—C16	118.94 (16)
H7A—C7—H7C	109.5	C19—C18—C17	120.95 (18)
H7B—C7—H7C	109.5	C19—C18—H18	119.5
C5—C8—H8A	109.5	C17—C18—H18	119.5
C5—C8—H8B	109.5	C20—C19—C18	120.19 (19)
H8A—C8—H8B	109.5	C20—C19—H19	119.9
C5—C8—H8C	109.5	C18—C19—H19	119.9
H8A—C8—H8C	109.5	C19—C20—C21	119.30 (19)
H8B—C8—H8C	109.5	C19—C20—H20	120.3
N10—C9—C14	122.13 (19)	C21—C20—H20	120.3
N10—C9—N1	113.83 (16)	C20—C21—C22	120.57 (19)
C14—C9—N1	124.04 (17)	C20—C21—H21	119.7
C9—N10—C11	117.88 (18)	C22—C21—H21	119.7
N10—C11—C12	123.88 (19)	C21—C22—C17	120.60 (18)
N10—C11—H11	118.1	C21—C22—H22	119.7
C12—C11—H11	118.1	C17—C22—H22	119.7
C11—C12—C13	118.26 (19)		
			
C9—N1—C2—O3	−6.7 (3)	C11—C12—C13—C15	−178.86 (18)
C16—N1—C2—O3	−178.23 (17)	C12—C13—C14—C9	−1.9 (3)
C9—N1—C2—O4	173.65 (15)	C15—C13—C14—C9	177.99 (16)
C16—N1—C2—O4	2.1 (2)	N10—C9—C14—C13	1.4 (3)
O3—C2—O4—C5	7.6 (3)	N1—C9—C14—C13	−178.34 (15)
N1—C2—O4—C5	−172.67 (14)	C2—N1—C16—C17	78.3 (2)
C2—O4—C5—C7	175.46 (17)	C9—N1—C16—C17	−93.7 (2)
C2—O4—C5—C8	−66.6 (2)	N1—C16—C17—C22	18.4 (3)
C2—O4—C5—C6	58.2 (2)	N1—C16—C17—C18	−163.51 (17)
C2—N1—C9—N10	−169.13 (16)	C22—C17—C18—C19	−0.7 (3)
C16—N1—C9—N10	2.5 (2)	C16—C17—C18—C19	−178.88 (19)
C2—N1—C9—C14	10.6 (3)	C17—C18—C19—C20	0.3 (4)
C16—N1—C9—C14	−177.75 (16)	C18—C19—C20—C21	−0.1 (4)
C14—C9—N10—C11	0.1 (3)	C19—C20—C21—C22	0.4 (3)
N1—C9—N10—C11	179.83 (16)	C20—C21—C22—C17	−0.9 (3)
C9—N10—C11—C12	−1.1 (3)	C18—C17—C22—C21	1.0 (3)
N10—C11—C12—C13	0.5 (3)	C16—C17—C22—C21	179.10 (19)
C11—C12—C13—C14	1.0 (3)		
